# Supplementary material for: Multiomics of GCN4-Dependent Replicative Lifespan Extension Models Reveals Gcn4 as a Regulator of Protein Turnover in Yeast
Source: Int J Mol Sci. 2023 Nov 10;24(22):16163. doi: 10.3390/ijms242216163 (PMC10671045; doi:10.3390/ijms242216163)
Supplement: Supplementary file 1 [file ijms-24-16163-s001.zip › ijms-2671982-supplementary.pdf]

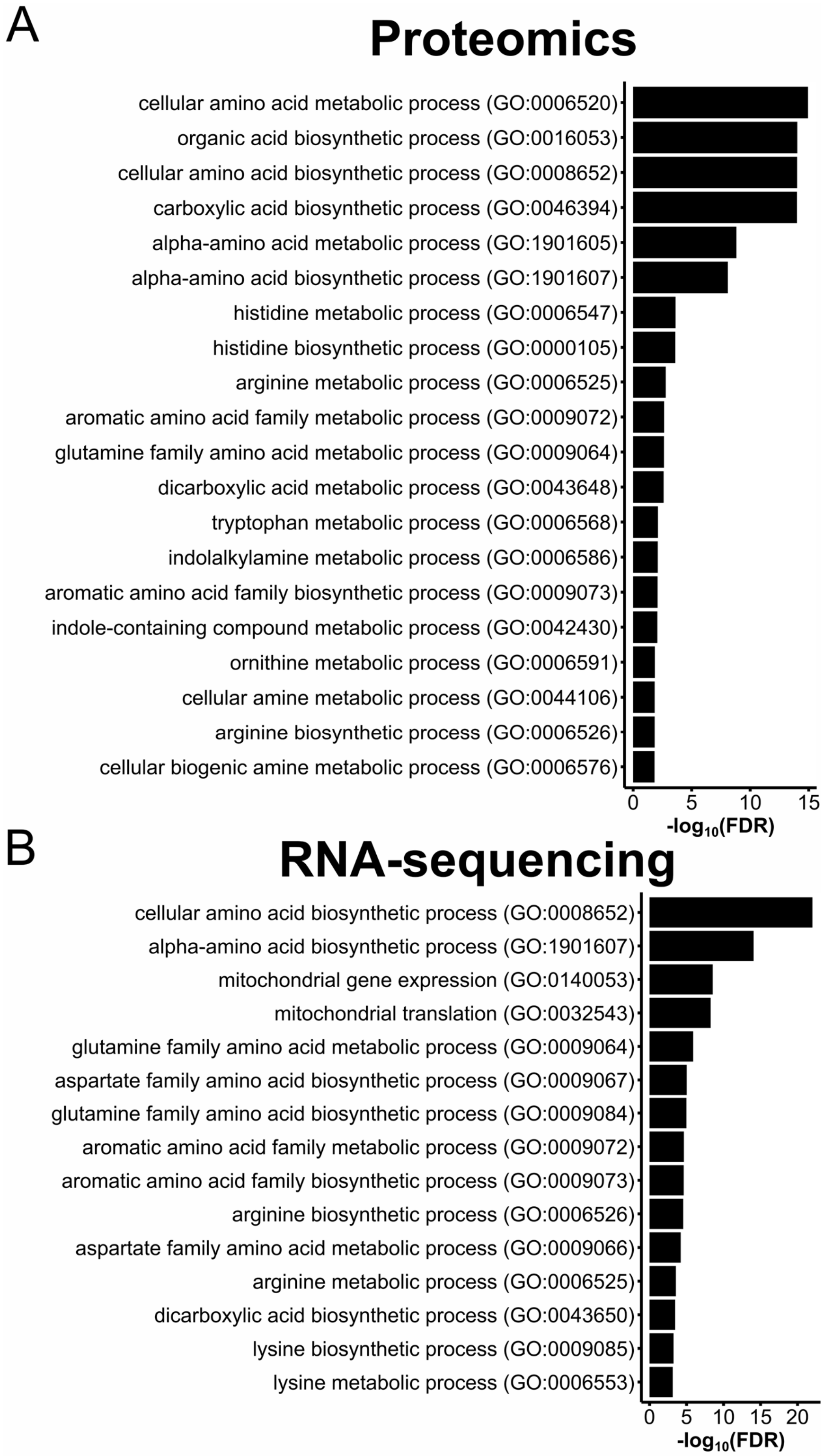

Figure S1: Over-represented ontology categories in the (A) proteomics and (B) RNAseq study of *GCN4*-dependent long-lived yeast.

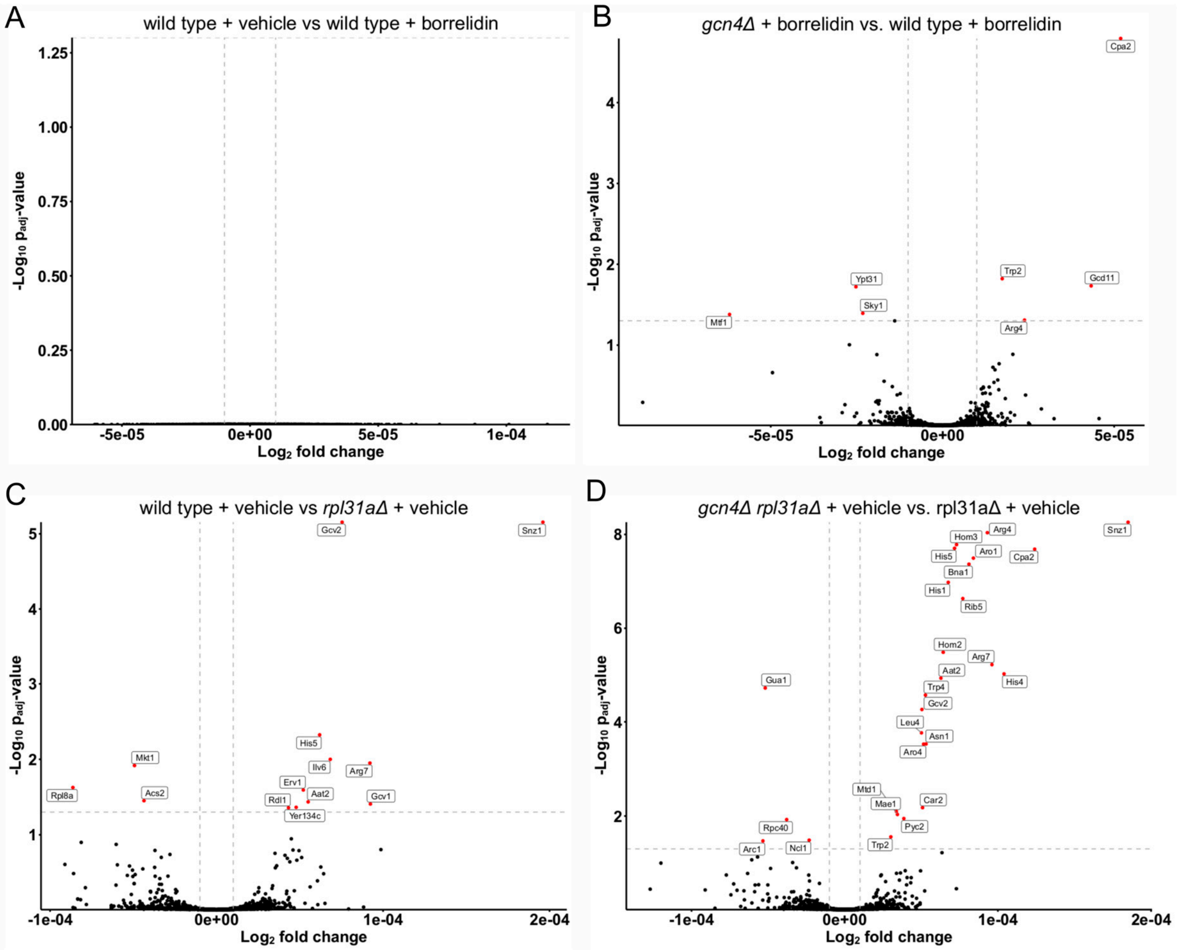

Figure S2: Single-condition comparisons of interest from the proteomics study (p_{adj} < 1e-4).

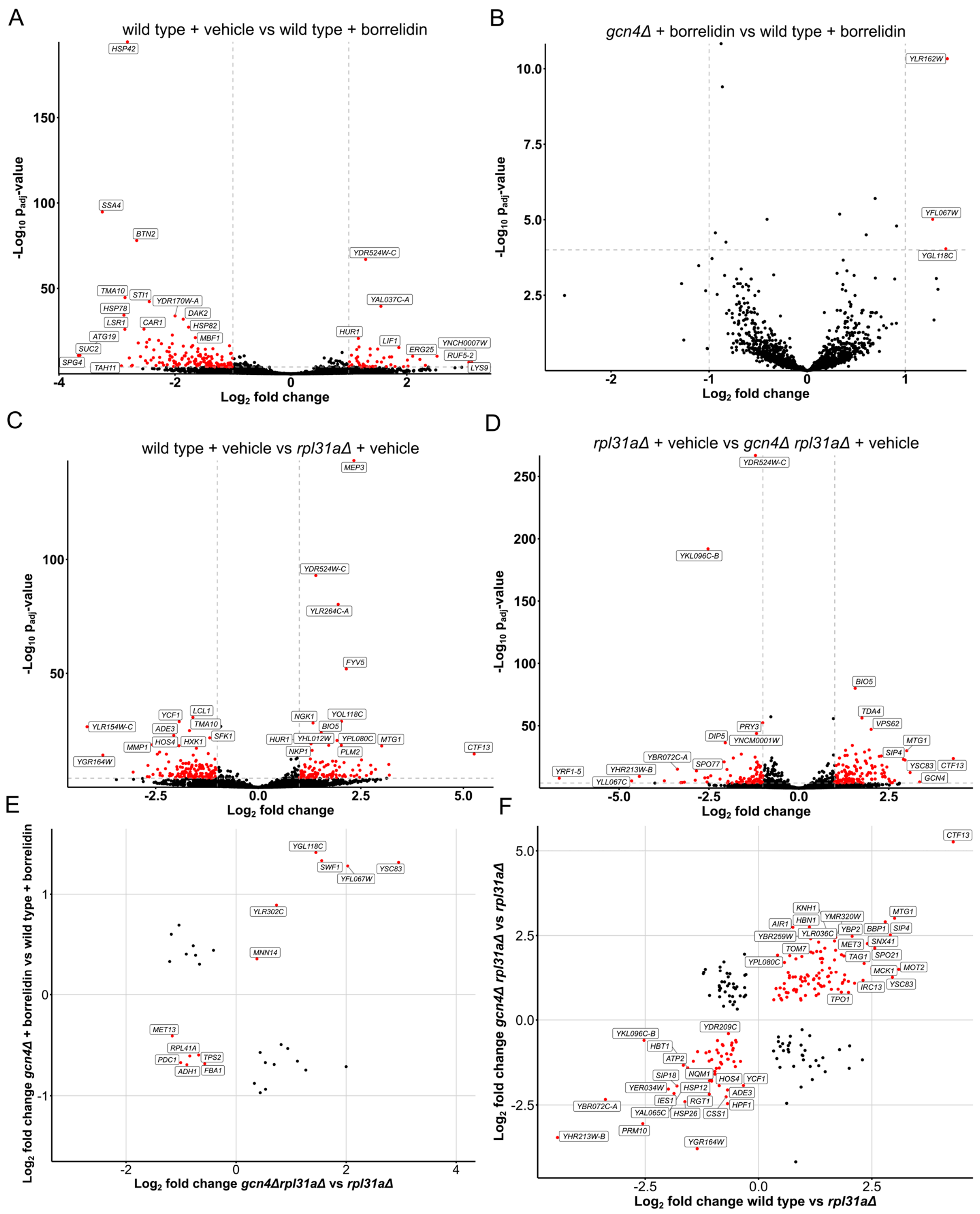

Figure S3: (A-D) Single-condition comparisons of interest from the RNAseq study. (E) Genes similarly differentially expressed in the wild type + borrelidin and *rpl31aΔ* yeast in comparison to their *gcn4Δ* counterparts (p_{adj} < 0.01). (F) Genes similarly differentially expressed in *rpl31aΔ* yeast in comparison to wild type and *gcn4Δ rpl31aΔ* yeast (p_{adj} < 0.01).

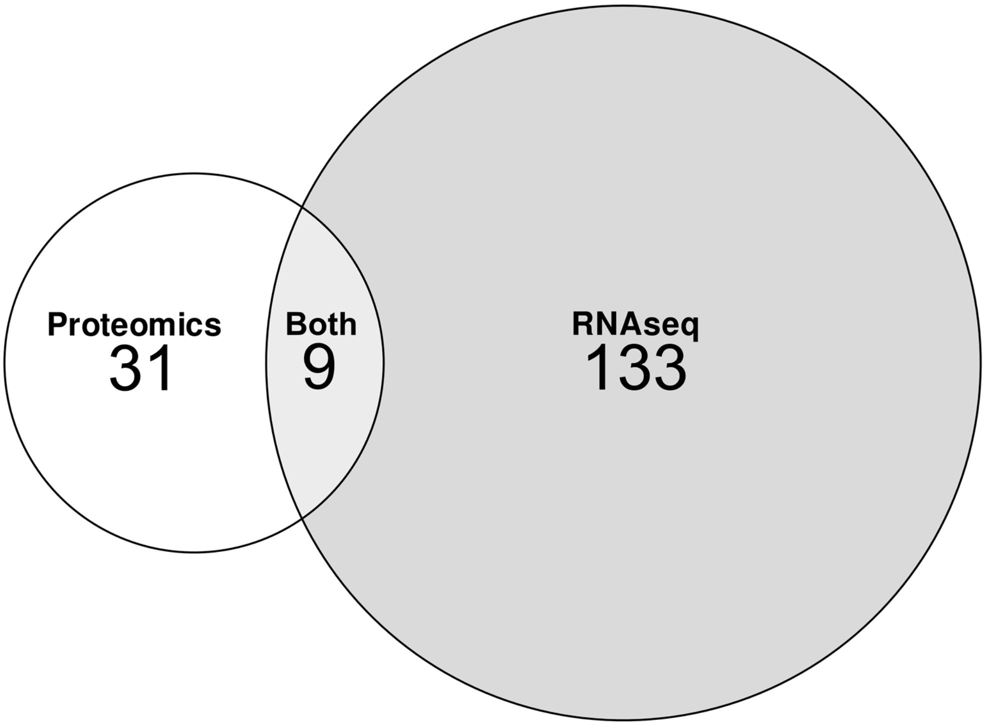

Figure S4: 142 of the significantly differentially expressed genes in the RNAseq study's linear model fit to Gcn4 translation ($p_{adj} < 0.05$) had protein abundance measurements in the proteomics study. Nine of these genes were recorded as having differential protein abundance from the proteomics study's linear model fit to Gcn4 translation.
